# Belowground Interaction in Tea/Soybean Intercropping Enhances Tea Quality by Improving Soil Nutrient Dynamics

**DOI:** 10.3390/plants14111691

**Published:** 2025-05-31

**Authors:** Tianqi Wang, Xiaoyu Mu, Erdong Ni, Qinwen Wang, Shuyue Li, Jingying Mao, Dandan Qing, Bo Li, Yuan Chen, Wenjie Chen, Cuiyue Liang, Hualing Wu, Xing Lu, Jiang Tian

**Affiliations:** 1 Root Biology Center, College of Natural Resource and Environment, Guangdong Engineering Technology Research Center of Low Carbon Agricultural Green Inputs, South China Agricultural University, Guangzhou 510642, China; wtq812@scau.edu.cn (T.W.); 15918645684@163.com (X.M.); caerwang@live.cn (Q.W.); lsy15082592313@163.com (S.L.); cloud09321@foxmail.com (J.M.); liangcy@scau.edu.cn (C.L.); jtian@scau.edu.cn (J.T.); 2 Tea Research Institute, Guangdong Academy of Agricultural Sciences, Guangdong Key Laboratory of Tea Plant Resources Innovation & Utilization, Guangzhou 510640, China; nierdong@gdaas.cn (E.N.); qdd8805@163.com (D.Q.); boniek0517@163.com (B.L.); 3 Grape and Wine Research Institute, Guangxi Academy of Agricultural Sciences, Nanning 530007, China; chenyuan313@163.com; 4 Cash Crops Research Institute, Guangxi Academy of Agricultural Sciences, Nanning 530007, China; cenwenji1030@163.com

**Keywords:** tea/soybean intercropping, tea quality, soil fertility, soil enzyme activity, belowground interactions

## Abstract

Although tea (*Camellia sinensis*)/soybean (*Glycine max*) intercropping is widely applied in tea gardens, the underlying mechanisms driving tea quality promotion remain largely unclear. This study explores the effects of intercropping on tea quality, soil nutrient availability, and soybean growth and analyzes their mutual relationship. Field experiments revealed that intercropping increased tea leaf water extracts, polyphenols, and amino acids by 4.36–8.99%, 14.76–15.23%, and 14.73–16.36%, respectively, across two growth stages. Furthermore, intercropping boosted organic matter, available nitrogen (N), phosphorus (P), and potassium (K) in the tea rhizosphere. Enzyme activities, including acid phosphatase, alkaline phosphatase, urease, and β-glucosidase, were also elevated in tea/soybean intercropping. In soybean, shoot and root biomass, weight and number of nodules, and N, P, and K content increased over cultivation time. Correlation analysis showed that tea water extracts and polyphenols were positively linked to soil available P and alkaline phosphatase activities. Soybean root and nodule growth were correlated with soil N and P activation and tea water extracts, indicating that soybean-mediated underground interactions drive mineral nutrient mobilization in rhizosphere, further improving tea quality. This study provides mechanistic insights into tea/soybean intercropping, offering practical implications for sustainable tea cultivation practices.

## 1. Introduction

Tea (*Camellia sinensis*), as a beverage made from plant buds and leaves, has gained widespread popularity globally [1]. Based on diverse processing methods, tea beverages can be divided into white, green, yellow, oolong, black, and dark teas. White and green varieties undergo minimal to no fermentation, whereas yellow tea experiences partial fermentation. In contrast, oolong, black, and dark teas are subjected to extensive fermentation processes [2,3,4,5]. The varying degrees of oxidation during production create distinct flavor profiles and chemical compositions across different tea categories. Studies indicate that regular tea intake may help decrease the risk of cardiovascular disorders and certain cancers [6] while also enhancing dental health and supporting various bodily functions, including blood pressure regulation, weight management, antimicrobial effects, and nervous system protection [7,8,9]. Tea cultivation, with its enduring legacy in China, has ascended to a pivotal position in the global beverage market [10]. Since 2010, driven by escalating market demand and supportive policies, both the cultivation area and production of tea in China have exhibited increasing trends [11,12]. In 2023, global tea production reached 6.6 million tons, of which China contributed 3.55 million tons, representing 54% of the total [13]. This substantial contribution underscores the dominant position of China in global tea production.

Tea plants thrive in acidic soil. However, soil acidification significantly degrades soil nutrient availability, posing a critical challenge to sustainable tea cultivation, especially in China [14,15]. Among soil nutrients, nitrogen (N) is indispensable for tea plant growth and the synthesis of secondary metabolites such as polyphenols, amino acids, and vitamins, which are directly related to tea quality [16,17,18]. Similarly, phosphorus (P) and potassium (K) also play vital roles in regulating the development of tea plants and the color of tea leaves [19,20,21]. However, the overuse of chemical fertilizers and improper agronomic practices have exacerbated the degradation of acidic soil properties, leading to a marked reduction in nutrient availability and, consequently, adversely affecting both the yield and quality of tea [22,23,24]. Therefore, enhancing nutrient cycling efficiency in the soil–plant system is paramount to improve tea production.

Intercropping, an eco-friendly agricultural system, involves the simultaneous cultivation of two or more crops. Recognized for its benefits in alleviating soil acidification [25], improving soil fertility [26], and enhancing crop yield and quality [27], intercropping is considered a crucial strategy for sustainable agriculture [28,29]. In particular, when legumes are incorporated, the productivity of the intercropping system can be further enhanced through their efficient nutrient utilization [30,31], which has also been proven in acidic soil [32]. Legumes develop nodules by symbiotic interactions with rhizobia, facilitating the conversion of atmospheric N into available forms for plants [33]. During this process, the released N from legumes can be transferred into the rhizosphere and utilized by neighboring plants, thereby improving N use efficiency in the intercropping system [34,35]. Additionally, organic acids secreted by legumes effectively activate insoluble soil P and K, promoting their availability within the intercropping system [36,37]. Due to these advantages, tea/legume intercropping has been widely adopted in tea gardens, significantly increasing tea yield and quality [38]. However, the mechanisms underlying the effects of legumes on soil properties and the quality of intercropped tea remain poorly understood, limiting the optimization of this agroforestry practice.

Based on these findings, we hypothesize that tea/soybean (*Glycine max*) intercropping might enhance tea quality by improving the nutrient bioavailability in soil through legume-driven rhizosphere processes. In this study, we conducted a field experiment to investigate the effects of tea/soybean intercropping on tea quality during two critical growth periods. To elucidate the underlying mechanisms of soil fertility regulation under intercropping conditions, we assessed N, P, and K availability, as well as the activities of related enzymes in the rhizosphere soil. Furthermore, we comprehensively analyzed the relationship between legume growth, soil properties, and tea quality in an intercropping system. This study provides a theoretical basis for the application of tea/soybean intercropping to promote agricultural sustainability.

## 2. Results

### 2.1. Tea/Soybean Intercropping Improves Quality of Tea Leaves

**Figure 1:**
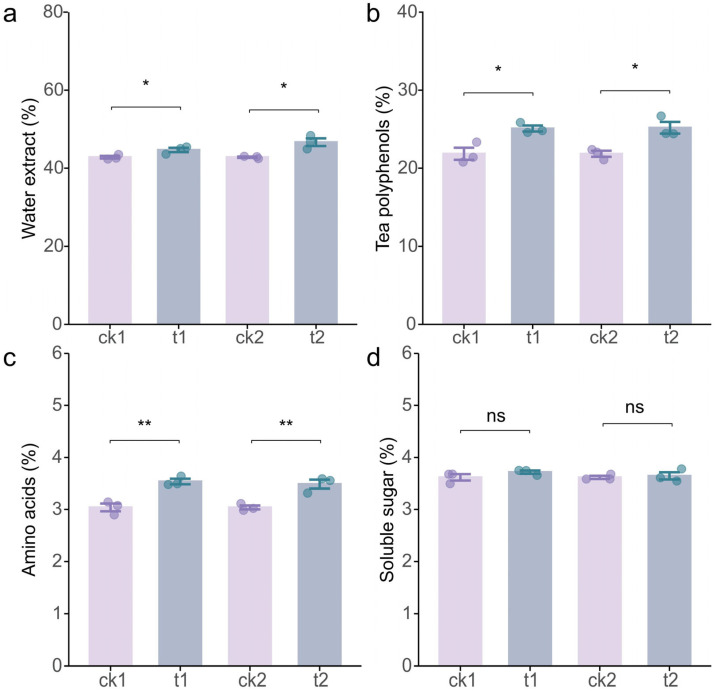
The effects of tea/soybean intercropping on tea quality. (**a**) Water extract content in tea leaves. (**b**) Tea polyphenol content in leaves. (**c**) Amino acid content in tea leaves. (**d**) Soluble sugar content in tea leaves. ck1, monoculture tea during the first growth stage; t1, intercropped tea during the first growth stage; ck2, monoculture tea during the second growth stage; t2, intercropped tea during the second growth stage. Data are presented as mean ± standard error, and dots represent individual values (n = 3 replicates). * and ** indicate significant differences by Student’s *t* test at *p* < 0.05 and *p* < 0.01, respectively. ns indicates no significant difference between treatments.

**Figure A1:**
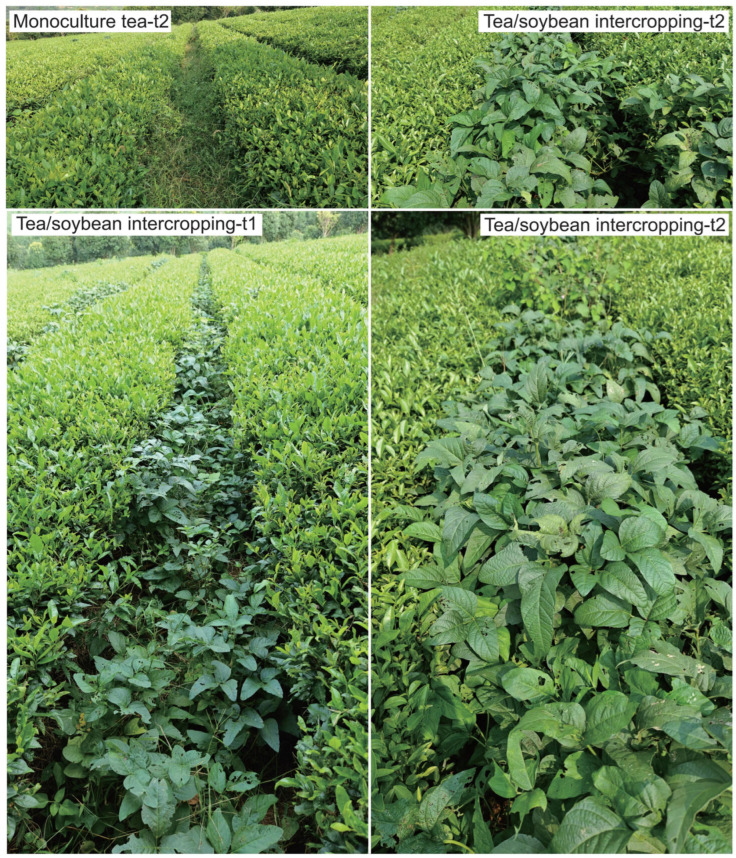
Photographs of tea/soybean intercropping and monoculture tea in 2023.

**Figure A2:**
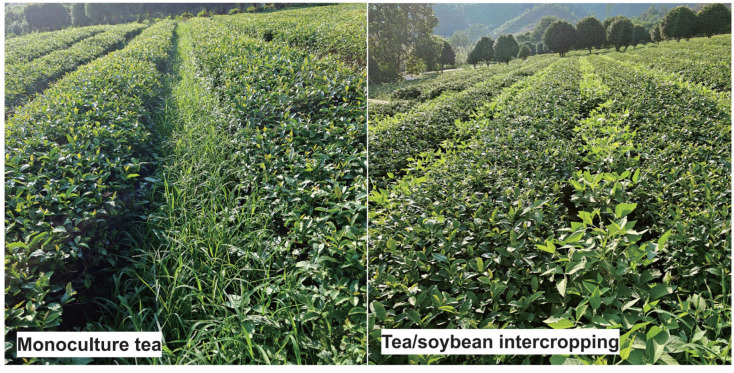
Photographs of tea/soybean intercropping and monoculture tea in 2024.

The tea/soybean intercropping experiments were conducted over two consecutive years (Figure A1 and Figure A2). The results demonstrated that intercropping significantly improved the quality of tea leaves compared to monoculture tea (Figure 1). Specifically, during the first growth stage, the contents of water extracts, tea polyphenols, and amino acids in intercropped tea leaves were significantly higher than those in monoculture tea leaves, with increases of 4.36%, 14.76%, and 16.36%, respectively. Similarly, during the second growth stage, these quality indicators were enhanced by 8.99%, 15.23%, and 14.73%, respectively (Figure 1a–c). However, intercropping with soybean did not exhibit a significant effect on the soluble sugar content in tea leaves (Figure 1d).

### 2.2. Tea/Soybean Intercropping Enhances Soil Properties of Tea Rhizosphere

**Figure 2:**
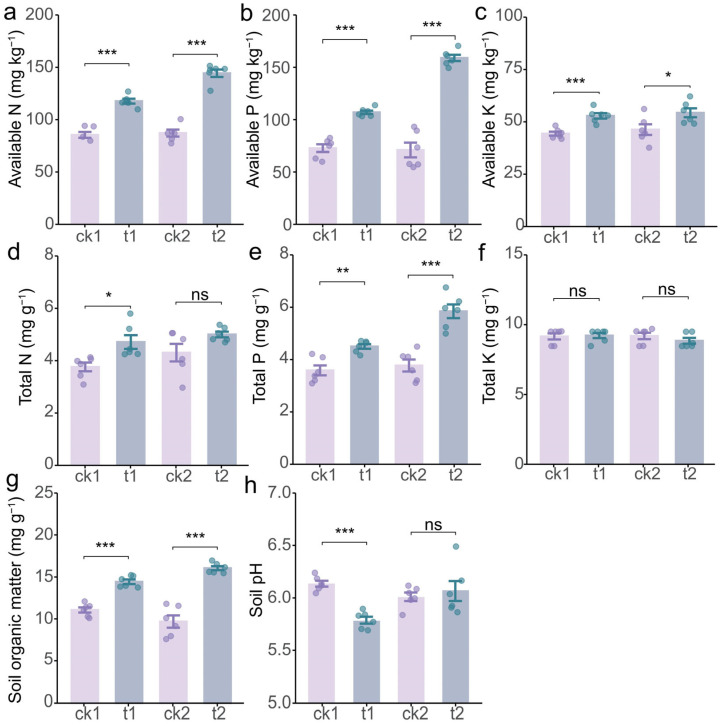
The effect of tea/soybean intercropping on soil fertility in the tea rhizosphere. (**a**) Available nitrogen (N) content. (**b**) Available phosphorus (P) content. (**c**) Available potassium (K) content. (**d**) Total N content. (**e**) Total P content. (**f**) Total K content. (**g**) Soil organic matter content. (**h**) Soil pH. ck1, monoculture tea during the first growth stage; t1, intercropped tea during the first growth stage; ck2, monoculture tea during the second growth stage; t2, intercropped tea during the second growth stage. The tops and bottoms of the boxes represent the 75th and 25th percentiles, respectively. The upper and lower whiskers extend 1.5× the interquartile range from the tops and bottoms of the boxes, respectively (n = 6 replicates). *, **, and *** indicate significant differences by Student’s *t* test at *p* < 0.05, *p* < 0.01, and *p* < 0.001, respectively. ns indicates no significant difference between treatments.

After assessing the soil fertility, our results showed that intercropping positively influenced soil fertility and nutrient availability in the tea rhizosphere (Figure 2). Specifically, tea/soybean intercropping significantly increased the contents of available N, available P, available K, total P, and soil organic matter (SOM) in the tea rhizosphere at both harvest times compared to monoculture tea (Figure 2a–c,e,g), represented by increases of 38.31%, 47.55%, 19.15%, 25.58%, and 30.5% at the first harvest time and 66.53%, 125.08%, 17.38%, 55.16%, and 65.7% at the second harvest time, respectively. Notably, the magnitude of these improvements exhibited a progressive enhancement with prolonged cultivation. Conversely, intercropping only elevated total N in the soil in the first growth stage, with minimal impact on total N and P in the second growth stage (Figure 2d,f). Additionally, soil pH in the tea rhizosphere was significantly reduced in the first growth stage, while this decrease was less pronounced in the second growth stage (Figure 2h).

**Figure 3:**
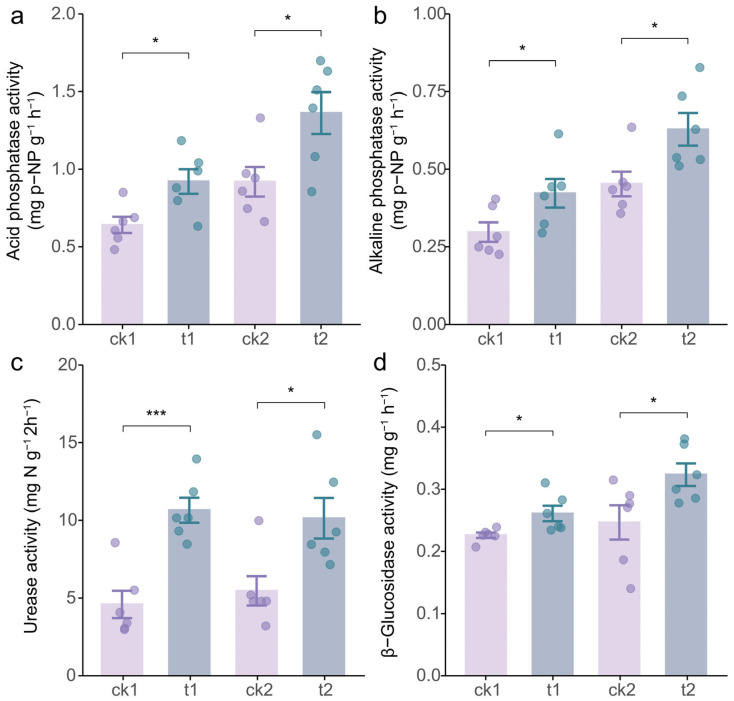
The effect of tea/soybean intercropping on soil enzyme activity in the tea rhizosphere. (**a**) Acid phosphatase activity. (**b**) Alkaline phosphatase activity. (**c**) Urease activity. (**d**) b-glucosidase activity. ck1, monoculture tea during the first growth stage; t1, intercropped tea during the first growth stage; ck2, monoculture tea during the second growth stage; t2, intercropped tea during the second growth stage. The tops and bottoms of the boxes represent the 75th and 25th percentiles, respectively. The upper and lower whiskers extend 1.5× the interquartile range from the tops and bottoms of the boxes, respectively (n = 6 replicates). * and *** indicate significant differences by Student’s *t* test at *p* < 0.05 and *p* < 0.001, respectively.

To reveal how tea/soybean intercropping enhanced soil nutrient availability, we investigated its impact on the activities of soil enzymes associated with nutrient cycling and activation in the tea rhizosphere. The results demonstrated that, compared to monoculture tea, tea/soybean intercropping significantly elevated the activities of acid phosphatase and alkaline phosphatase by 44.07% and 42.57% in the first growth stage and by 48.50% and 39.19% in the second growth stage, respectively (Figure 3a,b). Similarly, the activity of urease exhibited a marked elevation under intercropping, with increases of 132.31% and 85.57% at the two growth stages, respectively, relative to monoculture tea (Figure 3c). Furthermore, intercropping with soybean also enhanced the activity of β-glucosidase, an enzyme involved in soil carbon cycling (Figure 3d). Collectively, these findings revealed that tea/soybean intercropping enhances nutrient bioavailability by upregulating the activities of soil enzymes critical to nutrient cycling.

### 2.3. Effects of Different Stages on Growth and Nutrient Uptake of Intercropping Soybean

**Figure 4:**
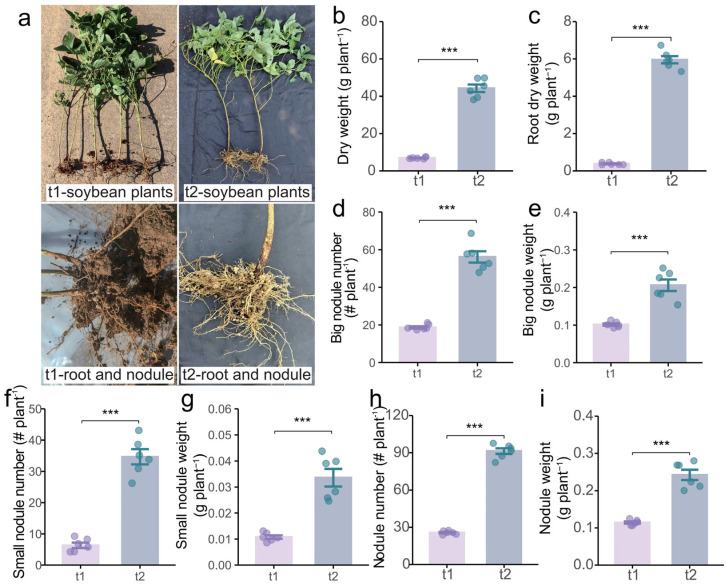
The effect of tea/soybean intercropping on soybean growth dynamics. (**a**) Photograph of intercropped soybean. (**b**) Shoot dry weight. (**c**) Root dry weight. (**d**) Big nodule number. (**e**) Big nodule weight. (**f**) Small nodule number. (**g**) Small nodule weight. (**h**) Total nodule number. (**i**) Total nodule weight. t1, intercropped tea during the first growth stage; t2, intercropped tea during the second growth stage. The tops and bottoms of the boxes represent the 75th and 25th percentiles, respectively. The upper and lower whiskers extend 1.5× the interquartile range from the tops and bottoms of the boxes, respectively (n = 6 replicates). *** indicates a significant difference by Student’s *t* test at *p* < 0.001.

To comprehensively evaluate the effects of the intercropping system, we subsequently investigated the dynamic changes in soybean growth and nutrient uptake (Figure 4a). The results revealed that the biomass of both shoots and roots in the second growth stage were significantly higher than those in the first growth stage, with 5.9-fold and 16.1-fold increases, respectively (Figure 4b,c). Moreover, the number and weight of big nodules (diameter > 2 mm) in the second growth stage were 2-fold and 1-fold greater than those in the first growth stage (Figure 4d,e), and the number and weight of small nodules (diameter < 2 mm) also increased 4.4-fold and 2.1-fold, respectively (Figure 4f,g), further enhancing the total nodule number and weight (Figure 4h,i). Therefore, tea/soybean intercropping positively regulates soybean growth, particularly in the underground components.

**Figure 5:**
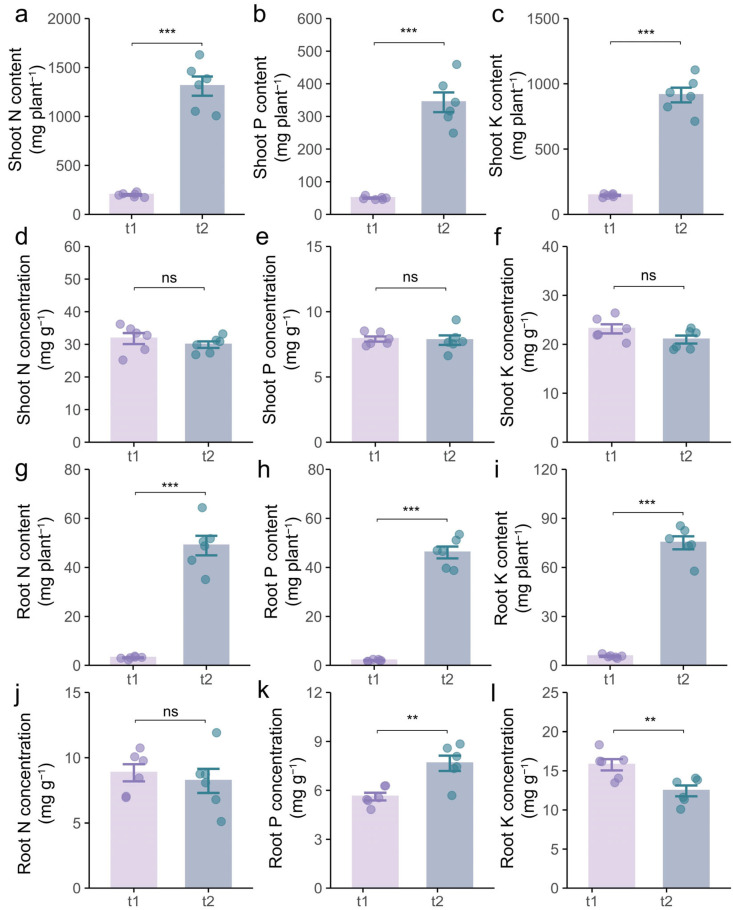
The effects of tea/soybean intercropping on soybean nutrient acquisition dynamics. (**a**) Shoot nitrogen (N) content. (**b**) Shoot phosphorus (P) content. (**c**) Shoot potassium (K) content. (**d**) Shoot N concentration. (**e**) Shoot P concentration. (**f**) Shoot K concentration. (**g**) Root N content. (**h**) Root P content. (**i**) Root K content. (**j**) Root N concentration. (**k**) Root P concentration. (**l**) Root K concentration. t1, intercropped tea during the first growth stage; t2, intercropped tea during the second growth stage. The tops and bottoms of the boxes represent the 75th and 25th percentiles, respectively. The upper and lower whiskers extend 1.5× the interquartile range from the tops and bottoms of the boxes, respectively (n = 6 replicates). ** and *** indicate significant differences by Student’s *t* test at *p* < 0.01 and *p* < 0.001, respectively. ns indicates no significant difference between treatments.

Meanwhile, the N, P, and K contents of shoots in the second growth stage were 5.6-fold, 5.9-fold, and 5.3-fold higher, respectively, than those in the first growth stage (Figure 5a–c). The corresponding nutrient contents in the roots exhibited even more pronounced increases, with 14.9-fold, 22.3-fold, and 12.6-fold elevations, respectively (Figure 5g–i). However, no significant differences were observed in the concentrations of N, P, and K in the shoot and root between the two growth stages (Figure 5d–f,j–l). In summary, tea/soybean intercropping mainly facilitates the accumulation of nutrients rather than the efficiency of nutrient uptake.

### 2.4. Relationship Among Soybean Growth, Soil Properties, and Tea Quality

**Figure 6:**
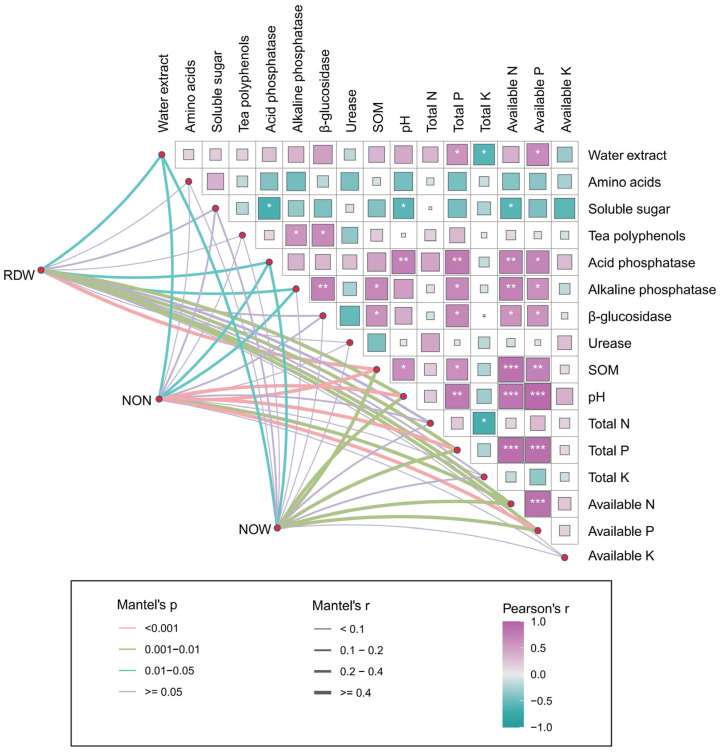
Soybean underground growth dynamics drive soil properties and tea quality. Pairwise comparisons of soil properties and tea quality are shown with a color gradient denoting Spearman’s correlation coefficient, with more positive values (purple) indicating stronger positive correlations and more negative values (green) indicating stronger negative correlations. The root and nodule growth dynamics were related to both soil properties and tea quality by partial (geographic distance corrected) Mantel tests. Edge width corresponds to the Mantel’s r statistic for the corresponding distance correlations, and edge color denotes the statistical significance based on 999 permutations. RDW, root dry weight; NON, nodule number; NOW, nodule weight.

To investigate the influence of soybean development on soil properties and tea quality, Mantel tests and Pearson’s correlation analysis were conducted. The results indicated that the water extract content exhibited a significant correlation with the total P (R = 0.58, *p* = 0.047) and available P contents (R = 0.66, *p* = 0.019) in the rhizosphere (Figure 6). Moreover, the tea polyphenol content demonstrated a strong positive correlation with the activity of alkaline phosphatase (R = 0.58, *p* = 0.045) and β-glucosidase (R = 0.65, *p* = 0.02) (Figure 6). These results suggest that adequate soil P availability is crucial for enhancing tea quality.

Additionally, the root dry weight (RDW), nodule number (NON), and nodule weight (NOW) of soybean showed significant positive correlations with SOM content, soil pH, total P, available N, and available P content, indicating that the development of soybean underground components may contribute to the activation of soil nutrients. Furthermore, RDW and NON were positively correlated with the activities of acid phosphatase and alkaline phosphatase, suggesting that soybean plays a beneficial role in organic P activation within the intercropping tea rhizosphere (Figure 6). Overall, the development of soybean underground parts might drive nutrient activation in the intercropped tea rhizosphere, thereby improving tea quality.

## 3. Discussion

Legumes are known to enhance nutrient activation and turnover in soil [39], improve fertilizer utilization efficiency [40], and promote sustainable agricultural development [34,41], thereby significantly benefiting neighboring plants in intercropping systems [42,43]. Specifically, tea/legume intercropping has been demonstrated to improve the quality and yield of tea [44,45]; however, the underlying mechanisms of these interspecific interactions remain poorly understood. This study elucidates the regulation of soybean on the quality and soil properties of intercropped tea, revealing the belowground synergistic process. The results demonstrate that tea/soybean intercropping significantly increased the content of water extracts, polyphenols, and amino acids in tea leaves (Figure 1) while also improving the availability of N, P, and K and the activity of related enzymes in the rhizosphere soil (Figure 2 and Figure 3). Further analyses suggest that these improvements may be attributed to the underground interactions between soybean and tea (Figure 4, Figure 5 and Figure 6). These findings provide a scientific basis for utilizing tea/soybean intercropping to promote sustainable tea production.

In the present study, the tea/soybean intercropping samples were collected in two stages, the podding stage and maturity stage of soybean. The two harvest stages of soybean represent distinct phases of belowground interactions in the intercropping system. During the podding stage, active symbiotic N fixation likely dominated the rhizosphere effects [46], enhancing soil total N availability and modulating pH (Figure 2d,h) to favor rhizobial colonization. In contrast, the maturity stage was characterized by substantial soybean litter deposition [47], which intensified rhizosphere modifications and led to greater increases in available N, available P, total P, organic matter, and acid/alkaline phosphatase activities of the tea plant rhizosphere compared to the first sampling (Figure 2a,b,e,g and Figure 3a,b). These temporal dynamics suggest that the growth phases of soybean differentially mediate soil nutrient mobilization. Optimizing intercropping schedules to align with these phases could further enhance nutrient utilize efficiency in tea cultivation systems.

The results indicate that tea/soybean intercropping significantly enhanced tea quality, with the concentrations of polyphenols and water extract showing a positive correlation with P availability and alkaline phosphatase activity in the rhizosphere soil (Figure 6). Similarly, Lin et al. [48] found that P deficiency adversely affects the sensory and biochemical quality of green tea leaves, including water extracts, polyphenols, flavonoids, and various amino acids. The underlying mechanism may be attributed to the pivotal role of P in chlorophyll synthesis [49,50], where improved P bioavailability increases photosynthetic efficiency in tea plants, thereby contributing to superior tea quality [51]. Furthermore, P is critical in regulating the metabolism and transport of beneficial elements and secondary metabolites in tea plants [52,53]. For instance, appropriate P application can balance the accumulation of amino acids and the metabolism of flavonoids in tea plants [54]. Cao et al. [55] demonstrated that optimal P concentrations enhance the absorption and translocation of selenium in tea, thereby increasing tea polyphenol content. Collectively, these findings underscore the indispensable role of adequate P supply in maintaining and improving tea quality.

In intercropping systems, the underground interactions between plants are pivotal in the activation of soil nutrients [56,57,58]. Consistently, the present results reveal that the development of soybean roots and nodules is closely associated with the SOM and availability of N and P in the tea rhizosphere (Figure 6), suggesting that belowground interspecific interactions in tea/soybean intercropping drive improvements in soil properties, thus enhancing tea quality. The enhanced bioavailability of N and P in the soil can be attributed to two primary reasons. On the one hand, soybean possesses the ability to convert atmospheric inert N into plant-available active N forms in the rhizosphere, which can be utilized by intercropped plants, thereby improving N utilization efficiency [35,39]. Moreover, the organic acids secreted by soybean roots, including citric acid, oxalic acid, and malic acid, can solubilize and chelate insoluble P, reducing P fixation and increasing P availability in the soil [59,60].

On the other hand, the current findings indicate that the activities of urease, acid and alkaline phosphatases, and b-glucosidase in the rhizosphere were significantly enhanced in the tea/soybean intercropping system (Figure 3a–c), while the growth of soybean roots and nodules was associated with the activity of phosphatases in the tea rhizosphere (Figure 6). The reason why intercropping elevated the two enzymes in the tea rhizosphere might be related to the microbial regulation by soybean. When intercropping with tea, soybean might release specific root exudates, such as organic acids, to recruit P-solubilizing microorganisms [61]. Subsequently, the microorganisms with capacity to secret acid phosphatase and alkaline phosphatase could activate the insoluble organic P for plant utilization [62]. Importantly, the observed increase in enzyme activities under intercropping reflects microbe-mediated nutrient cycling, driven by soybean root exudates and SOM. Specifically, acid/alkaline phosphatases were strongly correlated with available P, suggesting their role in P solubilization for tea plants, consistent with prior studies on legume-induced microbial *phoD* gene expression [63]. Moreover, urease activity aligned with soil N availability, likely due to rhizobium-enriched nodulation and subsequent urea hydrolysis [64]. Additionally, β-glucosidase responded to OM accumulation, reflecting microbial C demand [65]. While our current data support the role of soybean in nutrient–enzyme–microbe feedback, a metagenomic analysis of microbial taxa would strengthen mechanistic clarity.

Interestingly, the soil pH was found to decrease in the first growth stage of tea/soybean intercropping (Figure 2h). A possible reason is that the root exudates released by soybean, including organic acids and protons, may activate soil nutrients while simultaneously reducing soil pH [66,67,68]. In contrast, no significant difference in soil pH was detected between intercropping and monocropping systems in the second growth stage (Figure 2h). This shift may be related to the decomposition of soybean residues in long-term cultivation, which releases alkaline substances into the soil [69,70,71]. Overall, although tea/soybean intercropping leads to a decrease in soil pH in the short term, long-term intercropping may enhance the soil buffering capacity and mitigate soil acidification. Moreover, the SOM of the tea rhizosphere is significantly enhanced by intercropping, which could be also observed in previous studies [72,73]. The observed SOM elevation in tea-soybean intercropping primarily stems from soybean-mediated processes, including root exudates that stimulate microbial activity [74], continuous input of soybean root residues that decompose into stable organic carbon [75], and enhanced microbial biomass driven by symbiotic nitrogen-fixing rhizobia [45]. Importantly, our field trials strictly controlled the consistent organic fertilization application rate in the intercropping and monoculture of tea plants, which confirms that the SOM accumulation was solely due to intercropping effects, implying that our current data on legume intercropping strongly support this natural SOM enrichment process. As humic substances (HSs) typically constitute a large portion of stable SOM [76], HSs might also contribute to the improved nutrient availability and tea quality [77]. Future works may consider quantifying the contribution of HS in tea/soybean intercropping.

## 4. Materials and Methods

### 4.1. Plant Materials and Site Description

The soybean cv. Guixia 3 and 10-year-old plants of the tea cv. Bizaoxiang were used in this study. The experiment was performed in the Que Ming Chun Organic Tea Garden, Zhaoping County, Hezhou City, Guangxi Zhuang Autonomous Region (24.17° N, 110.81° E). The annual average temperature was 19.8 °C, and the annual average rainfall was about 2046 mm. The soil was classified as Ultisol. The soil properties of the experimental field are presented in Table A1.

### 4.2. Experimental Design and Sampling

The experiment included two cropping systems, tea/soybean intercropping and monoculture tea. Specifically, tea plants were spaced 110 cm apart. In intercropping treatments, two rows of soybeans with a 25 cm inter-row distance were planted between tea plants in April 2023 and April 2024, respectively (Figure A1 and Figure A2). In the tea plantation, organic fertilizers were applied at a rate of 7.5 t ha^−1^, consisting of peanut residue/sheep manure/sawdust = 1:1:1, before tea planting. The soybean received no fertilizer application. The soybean was direct-seeded. The tea plants were 10 years old, and were cut seedlings for transplanting. Before sowing, soybean seeds were inoculated with rhizobia as previously described [78]. Each hole contained three soybean seeds. The treatments were replicated in 6 randomized blocks. Each treatment plot in every block contained three rows and six tea plants in each row.

Samples of tea plants, soybeans, and rhizosphere soil were collected in the podding stage (July, 85 d after soybean germination) and maturity stage (August, 110 d after soybean germination) of soybean, representing the critical stage of symbiotic N fixation [46] and the stage of abundant soybean litter [47], respectively. The tea plants were totally harvested across four treatments: monoculture in July (ck1), intercropping in July (t1), monoculture in August (ck2), intercropping in August (t2). The soybeans were totally harvested in two treatments: intercropping in July (t1) and intercropping in August (t2). Specifically, six tea plants and soybean plants were randomly collected from each plot. Two fully expanded leaves with one single bud from the tea plant top were collected as fresh leaf samples. The soil sampling was conducted in the two harvest stages. The soil sampling locations were central between the tea and soybean rows in intercropping systems and the same locations in monoculture tea. The soil samples were collected using a stainless-steel auger (3 cm inner diameter) and conducted to a 20 cm depth at six spatially distributed points per treatment. Each soil sample was homogenized, sieved (<2 mm), and divided into two parts: one was stored at −20 °C for soil enzyme activity analysis, while the other was air-dried for the determination of soil chemical properties. Soybean leaves, roots, and nodules were separately harvested. Their dry weights were determined, along with nodular traits: number of small (≤2 mm diameter) and big (>2 mm diameter) nodules, total nodule number, weights of small and big nodules, total nodule weight, and nodule fresh weight.

### 4.3. Determination of Tea Quality

The tea leaves from two random blocks were combined into one replicate, and each sample contained three replicates. The water extract content of tea was measured by the weight method [79]. Briefly, the ground samples were extracted in boiled distilled water for 45 min. Then, the extracted infusion was placed in a weighed evaporation dish to evaporate to dryness over a heated water bath. The water extract content was calculated with the weight difference of the evaporation dish before and after evaporation. Tea polyphenol content was determined by the colorimetry method [80]. Briefly, 1 mL of the extracted solution was infused with 19.0 mL mixture (distilled H_2_O 4.0 mL, dyeing solution 5.0 mL, buffer 15 mL), and then the absorbance was measured at 540 nm. Free amino acid levels were determined by the ninhydrin dyeing method. Briefly, 1 mL of the extracted solution was infused with 0.5 mL of ninhydrin solution (2%) and 0.5 mL of phosphate buffer (pH = 8.0). Then, the mixture was heated for 15 min. After cooling to room temperature, the mixture was diluted with 25 mL water, and the absorbance was measured at 570 nm [81]. Soluble sugars were determined by the anthrone colorimetric method [48]. Briefly, 1 mL of the extracted solution and 8.0 mL of anthrone reagent (1.0 mg/mL anthrone–pure sulfuric acid solution) were heated at 100 °C for 7 min, and finally, the absorbance was measured at 620 nm. Glucose was used to draw a standard curve.

### 4.4. Measurement of Soil Fertility

Soil pH was determined using a pH meter in a 1:2.5 (*v*/*v*) soil-to-water suspension after 30 min equilibration. The SOM was quantified by K_2_Cr_2_O_7_–H_2_SO_4_ oxidation at 170–180 °C for 5 min, followed by titration with ferrous sulfate (FeSO_4_) to determine residual Cr_2_O_7_^2−^ [82]. For soil total N content, the soil was digested with concentrated H_2_SO_4_ and catalyst (CuSO_4_:Se, 10:1 ratio) using the Kjeldahl method, followed by steam distillation and titration with 0.01 M HCl [83]. For soil total P content, the soil was digested with H_2_SO_4_–HClO_4_ (9:1, *v*/*v*) at 200 °C and then measured by molybdenum blue colorimetry at 880 nm [84]. For soil total K, the soil was fused with NaOH at 720 °C for 15 min, dissolved in 0.5 M HCl, and quantified by flame photometry [85]. For soil available N content, the alkaline diffusion method was performed with 1 M NaOH to release NH_3_, trapped in 2% boric acid and titrated [86]. Soil available P content was extracted using the Bray II method (0.03 M NH_4_F + 0.025 M HCl) and analyzed by the ascorbic acid–molybdate method [87]. Soil available K content was extracted with 1 mol L^−1^ neutral NH_4_OAc (pH 7.0) and measured via flame photometry [88].

### 4.5. Determination of Soil Enzyme Activities

Soil enzyme activities were measured with fresh soil samples. Soil acid/alkaline phosphatase activity was determined using p-nitrophenyl phosphate (pNPP) as the substrate. Briefly, 1 g of fresh soil was incubated with 0.2 mL toluene and 4 mL buffer (buffer pH 6.5 for acid phosphatase activity and pH 11 for alkaline phosphatase activity) and 1 mL 50 mM pNPP at 37 °C for 1 h. Reactions were terminated with 1 mL 0.5 M CaCl_2_ and 4 mL 0.5 M NaOH solution, and then samples were centrifuged and supernatant absorbance was measured at 400 nm. Activity was calculated as μg p-nitrophenol (pNP) released per gram soil per hour using a standard curve [89,90].

Soil urease activity was determined using urea as the substrate. Briefly, 1 g of fresh soil was incubated with 10 mL citrate buffer (pH 6.7), 1 mL toluene, and 10 mL 10% urea solution at 37 °C for 1 h. Reactions were terminated by adding 10 mL KCl solution (2 M), and then samples were centrifuged, and supernatant ammonium-N was quantified via colorimetry (indophenol blue method) at 630 nm. Activity was calculated as μg NH_4_^+^-N released per gram of soil per hour using a standard curve [89,91].

β-glucosidase activity was determined using a colorimetric method with p-nitrophenyl-β-D-glucopyranoside (pNPG) as the substrate, adapted from [89,92]. Briefly, 1 g fresh soil was weighed into 50 mL centrifuge tubes. And 4 mL acetate buffer (pH 6.0) and 1 mL pNPG solution were added into the tubes. Samples were reacted at 37 °C for 1 h with gentle shaking. Reactions were stopped with 1 mL 0.5 M CaCl_2_ and 4 mL 0.1 M Tris buffer. Samples were centrifuged at 3000 rpm for 5 min, and supernatant absorbance was measured at 410 nm. Values were calculated using a standard curve prepared with pNP standards. The β-glucosidase activity was calculated as mg pNP released per gram of soil per hour.

### 4.6. Measurement of Nutrient Acquisition of Soybean Plants

The soybean plant samples were ground and digested with H_2_SO_4_-H_2_O_2_ reagent, and the N and P concentrations in the supernatant were analyzed using a continuous flow analyzer (Series SA1100, SKALAR, Breda, The Netherlands) [93]; K analysis was performed by flame photometry [94]. The N, P, and K contents of the plants were subsequently calculated as follows:

N content (mg plant^−1^) = Plant N concentration (mg g^−1^) × Plant dry weight (g plant^−1^)
P content (mg plant^−1^) = Plant P concentration (mg g^−1^) × Plant dry weight (g plant^−1^)
K content (mg plant^−1^) = Plant K concentration (mg g^−1^) × Plant dry weight (g plant^−1^)

### 4.7. Statistical Analysis

Data were analyzed and visualized using the “dplyr” and “ggplot2” packages in R software (version 4.4.3). Differences between means were analyzed by Student’s *t* test using R software. The correlations between the soybean growth, tea quality, and soil properties were assessed using Spearman’s correlation and Mantel tests with the “vegan” and “ggcor” packages in R software.

## 5. Conclusions

Tea/soybean intercropping demonstrates significant potential to enhance tea quality, concurrently improving soil properties and nutrient availability. Our findings demonstrate that soybean root development and nodulation act as key mediators of nutrient mobilization in the tea rhizosphere, directly elevating tea quality parameters. Future research should isolate specific root exudates and microbial consortia to further elucidate causal mechanisms. This study provides valuable insights into the ecological and agricultural significance of tea/soybean intercropping, contributing to the advancement of sustainable agricultural practices.

## Data Availability

The datasets generated and analyzed during the current study are available from the corresponding author on reasonable request.

## Abbreviations

The following abbreviations are used in this manuscript:

N	Nitrogen
P	Phosphorus
K	Potassium
SOM	Soil organic matter
HS	Humic substance
pNPP	p-nitrophenyl phosphate
pNP	p-nitrophenol
pNPG	p-nitrophenyl-β-d-glucopyranoside

## Appendix A

**Table A1:** Soil properties of the experimental field.

pH	Available N	Available P	Available K	Total N	Total P	Total K
	mg·kg^−1^			mg·g^−1^		
5.83	79.1	59.56	42.32	4.67	5.92	9.45
